# Impact of weather parameters on Alternaria leaf spot of soybean incited by *Alternaria alternata*

**DOI:** 10.1038/s41598-022-10108-z

**Published:** 2022-04-12

**Authors:** R. K. Fagodiya, Amit Trivedi, B. L. Fagodia

**Affiliations:** Department of Plant Pathology, Maharana Pratap University of Agriculture and Technology, Udaipur, Rajasthan 313 001 India

**Keywords:** Plant sciences, Environmental sciences

## Abstract

Weather attributes play a crucial role in the infection process and spread of pathogen. Alternaria leaf spot incited by *Alternaria alternata* is most destructive disease of soybean appeared in southern and eastern parts of Rajasthan as well as India. The effect of various weather parameters along with different date of sowing on the development of Alternaria leaf spot in susceptible soybean cultivar RKS-24 was investigated during *Kharif* season 2018 and 2019. The various weather factors viz*.,* temperature, relative humidity and rainfall under inoculated conditions and with staggered dates of sowing were taken to observe effect on disease progression and their effect on seed yield. The maximum increase in disease severity (57.82 and 58.22%) and AUDPC (389.45 and 394.42) recorded in crop sown on 18th June (inoculated on 8th July). Lowest disease severity (39.80 and 38.50%) and AUDPC (266.18 and 259.18) were observed during 39–43th standard meteorological week (September, 24–October, 28) in year 2018 and 2019, respectively. Maximum seed yield (1699 kg ha^−1^) was recorded in plants sown on 9th July, while, lowest seed yield was recorded in plants sown on 18th June with 1441.20 kg ha^−1^. The trend of disease severity and AUDPC value decreased from early sowing to late sowing (18th June–9th July). Major reasons were fluctuations in temperature, rainfall and relative humidity. It was also observed that the soybean plants for Alternaria leaf spot disease in early sowing were predisposed and so farmers should be advised to practice delayed sowing of soybean crop.

## Introduction

Soybean (*Glycine max* L.) contains highest protein (40–42%) among cultivated pulses. It also contains 18–20% oil content with calcium, iron and glycine^1^ and used as protein concentrates, vegetable oil for humans and a high-quality animal feed^2^. In India, the cultivation of soybeans started after 1960 and very quickly it attends premium position in pulses growing areas due to high demand, price and an industrial crop. However, the venerability of crops to different fungal infections is major constraints for growers and traders, which adversely affects their marketing in international trades. Presently, the United States is a major producer of soybean worldwide and contributes about 35% (342 MT) of total global production^3^. The production of soybean is forecasted to grow by 55%, in contrast with the 100–110% estimated demand increase by 2050^4^. Therefore, increasing overall productivity through implementation of suitable management technologies of the disease and pests and other aspects is a research priority from a food and nutritional security goal. The various diseases, pests and weeds are the major threat for soybean crop production as well as the changing scenario of weather parameters are also increased their incidence possibility. In this context, the study of relationship between climatic conditions and progression of disease severity is an important aspect. The average annual yield losses due to soybean diseases in the United States are estimated to be approximately 11%^5^. The phyllo sphere region of plants is a dynamic ecosystem inhibited by specific fungi, bacteria and yeasts. Their activity is related to various interactions between the biotic and abiotic factors of the environment^6^. Abiotic factors include temperature, pH, relative humidity, light intensity, rainfall etc. whereas biotic factors include pest and other microorganism like fungi, bacteria, virus and yeasts will complete with pathogenic species this phenomenon called antagonistic activity^7^. *Alternaria alternata* causes leaf spot and leaf blight diseases in a series of agricultural crops including soybean, which causes a significant losses^8^. Alternaria leaf spot, caused by the necrotrophic fungus *A. alternata*, is one of the most destructive foliar diseases of soybean growing areas of world and their pathogenicity also increased in present changing climate scenario. In India, Alternaria leaf spot has been reported in soybean by various scientist^9,10^. The disease infection is insignificant at early stages of growth, but it caused significant infection at later stage of plant growth. The disease incidence becomes very severe resulting in considerable yield loss. It appears broadly during first week of September when crop is in vegetative growth and manifests as brown necrotic spots with concentric rings on foliage. These spots coalesce and form large necrotic areas in advanced stages. Infected leaves eventually dry out and drop prematurely. A yellow halo is seen around the spots on leaves^11^. The management of this disease through chemicals are available but is not safe for humans, animals and caused environmental effects. Considering the importance of the crop, destructive nature of the pathogen, the management of these infecting crops is difficult due to unavailability of commercial resistant varieties against the disease. The disease progress curve, referred to as the signature of an epidemic, represents the integration of all the host, pathogen and environmental effects during the epidemic^12^. The Area Under Disease Progress Curved (AUDPC) decreased significantly for all different weather parameters. The correlation between AUDPC and weather parameters varied. AUDPC was strongly correlated with all the meteorological attributes. The value of AUDPC was negatively correlated to different weather attributes proving that the pathogen had a damaging effect on the crop attributes in oilseed crops^13^. The natural epidemics of Alternaria leaf spot are strongly influenced by environmental conditions and severe disease appears every year in India. Most of the Indian cultivars are susceptible to Alternaria leaf spot disease and no availability of resistant varieties in the market. Further, the aim of the present study, the knowledge about the time when pathogen attack, progression of disease and their relation to weather parameters is inevitable to be investigated for suitable management of disease through adjustment in sowing dates. One of the reasons would be insufficient information in the epidemiological aspects of this disease.

## Results

The progression of Alternaria leaf spot disease severity with weather conditions during *Kharif* season 2018 and 2019. The results revealed that, the per cent disease index increased from the appearance of the disease in field. However, in first two dates of sowing 18th June and 25th June appearance of disease was recorded on 16th July and 23rd July, while the disease appearance was delayed in other two dates of sown crop. The per cent disease index periodically increased with the time in all dates of sowing. From 16th July to 12th August the disease progression was slow among different date of observations in different dates of sowing. The maximum disease severity was recorded in early sown crop (18th June), while minimum disease severity was recorded in 9th July sown crop (Fig. 4).

### Effect of weather parameters on disease severity and seed yield during *Kharif,* 2018

**Figure 1:**
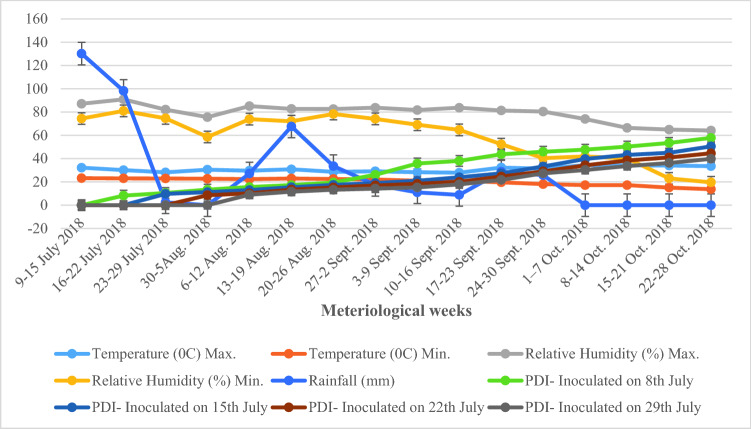
Progression of Alternaria leaf spot on soybean in relation to weather parameters in different dates of sowing under epiphytotic conditions during *Kharif* 2018.

The progression of disease was slow during the period between 16th July to 2nd September and during this period, the disease severity slightly increased from 8.21–26.11, 9.67–19.01, 8.54–16.90 and 9.0–14.20% in 18th June, 25th June, 2nd July and 9th July sown crops, respectively. During this period, the minimum and maximum temperatures were 22.0–32.2 °C and relative humidity 74.1–83.7% with 47.07 mm average rainfall. Following this period, the disease severity increased gradually. There was a sharp increase in disease severity (39.80–57.82%) observed during 24th September–28th October in all dates of sowing (Fig. 1).

The disease severity was positively correlated with maximum temperature, while it is negatively correlated with minimum temperature, minimum and maximum relative humidity and rainfall for all dates of sowing (Table 1). The predication equations explained 90.33, 94.57, 91.26 and 91.14% for disease development as influenced by the maximum and minimum temperature, relative humidity and rainfall in all dates of sowing (Table 2). On the basis of per cent disease severity, the maximum AUDPC was recorded as 389.45, when crop sown on 18th June. However, minimum AUDPC was recorded as 266.18, when crop sown on 9th July (Table 3). The maximum seed yield (1715.74 kg ha^−1^) was recorded in late sown crop 9th July and minimum seed yield was recorded (1461.11 kg ha^−1^) in early sown crop 18th June (Table 7).

**Table 1:** Correlation matrix between weather parameters and per cent disease severity in different date of sowing during *Kharif,* 2018.

S. No	Date of sowing	Per cent disease severity at different weather parameters				
		Temperature (°C)		RH (%)		Rainfall (mm)
		Maximum	Minimum	Maximum	Minimum	
1	18th June	0.555*	− 0.920**	− 0.760**	− 0.866**	− 0.647*
2	25th June	0.642**	− 0.954**	− 0.872**	− 0.913**	− 0.666**
3	02nd July	0.803**	− 0.980**	− 0.839**	− 0.915**	− 0.522*
4	09th July	0.838**	− 0.985**	− 0.929**	− 0.976**	− 0.681**

*and **Represent significant at 5 and 1%, respectively.

**Table 2:** Multiple regression equation for prediction of progress of Alternaria leaf spot severity at different dates of sowing in relation to weather parameters during *Kharif*, 2018.

S. No	Date of sowing	Regression equation	R^2^ value
1	18th June	Y = 81.595 + 0.412 T max. − 6.660 T min. + 0.903 RH max. + 0.0642 RH min. − 0.144 RF	0.9033
2	25th June	Y = 97.916 + 0.678 T max. − 4.937 T min. − 0.068 RH max. + 0.215 RH min. − 0.0977 RF	0.9457
3	02nd July	Y = 90.786 + 0.713 T max. − 4.781 T min. − 0.0833 RH max. + 0.203 RH min. − 0.0466 RF	0.9126
4	09th July	Y = 91.772 + 0.550 T max. − 5.900 T min. + 0.119 RH max. + 0.330 RH min. − 0.0306 RF	0.9114

**Table 3:** Progression of Alternaria leaf spot on soybean in relation to weather parameters in different dates of sowing under epiphytotic conditions during *Kharif* 2018.

S.No	Standard week	Meteorological weeks	Temperature (°C)		Relative humidity (%)		Rainfall (mm)	AUDPC*			
			Max	Min	Max	Min		Inoculated on			
								8th July	15th July	22th July	29th July
1	28	9–15 July 2018	32.2	23.2	87.1	74.4	130.2	0.00	0.00	0.00	0.00
2	29	16-22July 2018	30.1	23.0	90.9	81.0	98.2	31.74	0.00	0.00	0.00
3	30	23–29 July 2018	28.2	22.9	82.1	74.6	2.4	65.31	33.85	0.00	0.00
4	31	30-5Aug. 2018	30.5	22.7	75.6	58.6	0.0	83.13	73.15	29.89	0.00
5	32	6–12 Aug. 2018	29.6	22.4	85.1	73.9	27.2	100.98	84.67	65.42	28.50
6	33	13–19 Aug. 2018	30.8	22.9	82.7	72.1	67.6	114.66	99.37	82.22	71.96
7	34	20–26 Aug. 2018	28.6	22.5	82.6	78.3	33.6	128.28	114.91	99.89	86.77
8	35	27–2 Sept. 2018	29.1	22.0	83.7	74.1	17.4	159.43	127.44	112.35	96.01
9	36	3–9 Sept. 2018	28.3	21.0	81.7	69.1	11.0	216.55	140.04	123.38	102.94
10	37	10–16 Sept. 2018	27.9	21.0	83.7	64.7	8.8	258.16	157.33	134.23	115.71
11	38	17–23 Sept. 2018	32.3	19.6	81.3	52.4	28.8	286.09	179.62	155.96	138.32
12	39	24–30 Sept. 2018	31.3	18.1	80.4	40.4	25.8	313.74	212.07	187.50	171.50
13	40	1–7 Oct. 2018	34.4	17.2	74.0	41.9	0.0	327.46	255.12	221.34	201.39
14	41	8–14 Oct. 2018	34.2	17.2	66.4	40.1	0.0	342.86	289.94	254.28	223.20
15	42	15–21 Oct. 2018	34.0	15.1	64.9	23.0	0.0	363.13	308.60	277.97	244.34
16	43	22–28 Oct. 2018	33.5	13.7	64.1	19.6	0.0	389.45	334.25	299.46	266.18

*Mean of three replications, Inoculation started 21 days after sowing of each date. *Observation started 7th days after inoculation and at weekly intervals, 1st sown-18th June, 2nd–25th June, 3rd–02th July, 4th–09th July 2018.

### Effect of weather parameters on disease severity and seed yield during *Kharif,* 2019

**Figure 2:**
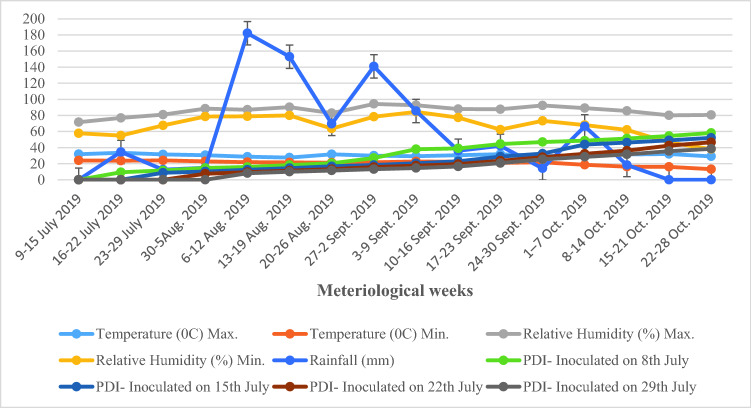
Progression of Alternaria leaf spot on soybean in relation to weather parameters in different dates of sowing under epiphytotic conditions during *Kharif* 2019.

The progression of disease was slow during the period between 16th July to 2nd September and during this period, the disease severity increased from 9.41 to 27.12, 8.76–18.41, 7.54–15.80 and 8.15–13.20% in 18th June, 25th June, 2nd July and 9^th^ July sown crops, respectively. In this period, the minimum and maximum temperatures were 21.3–33.6 °C and relative humidity 55.0–94.3% with 76 mm average rainfall. Following this the disease severity increased gradually. The sharp increase in disease severity (38.50–58.22%) was observed during 24th September–28th October in all dates of sowing (Fig. 2).

The disease severity was negatively correlated with minimum temperature for all dates of sowing. But disease severity was negatively correlated with maximum, minimum relative humidity and rainfall in crop sown as 2nd July and 9th July (Table 4). The predication equations explained 85.76, 93.07, 94.14 and 89.50% for disease development as influenced by the maximum and minimum temperature, relative humidity and rainfall in all dates of sowing (Table 5). On the basis of per cent disease severity, the maximum AUDPC was recorded as 394.42, when crop sown on 18th June. However, minimum AUDPC was recorded as 259.18, when crop sown on 9th July (Table 6). The maximum seed yield (1683.33 kg ha^−1^) was recorded in late sown crop (9th July) and minimum seed yield was recorded (1421.29 kg ha^−1^) in early sown crop 18th June (Table 7).

**Table 4:** Correlation matrix between weather parameters and per cent disease severity in different date of sowing during Kharif, 2019.

S. No	Date of sowing	Per cent disease severity at different weather parameters				
		Temperature (°C)		RH (%)		Rainfall (mm)
		Maximum	Minimum	Maximum	Minimum	
1	18th June	− 0.166	− 0.796**	0.335	− 0.308	− 0.254
2	25th June	− 0.164	− 0.910**	0.201	− 0.428	− 0.270
3	02nd July	0.279	− 0.902**	− 0.539*	− 0.840**	− 0.650**
4	09th July	0.348	− 0.881**	− 0.536*	− 0.820**	− 0.828**

*and **Represent significant at 5 and 1%, respectively.

**Table 5:** Multiple regression equation for prediction of progress of Alternaria leaf spot severity at different dates of sowing in relation to weather parameters during *Kharif*, 2019.

S. No	Date of sowing	Regression equation	R^2^ value
1	18th June	Y = − 109.803 + 1.258 T max. − 3.653 T min. + 2.448 RH max. − 0.386 RH min. − 0.095 RF	0.8576
2	25th June	Y = − 7.476 + 0.961 T max. − 4.882 T min. + 1.240 RH max. + 0.010 RH min. − 0.068 RF	0.9307
3	02nd July	Y = − 16.179 + 0.398 T max. − 3.797 T min. − 1.405 RH max. + − 0.235 RH min. − 0.050 RF	0.9414
4	09th July	Y = − 33.197 + 0.574 T max. − 3.116 T min. + 1.386 RH max. − 0.315 RH min. − 0.028 RF	0.8950

**Table 6:** Progression of Alternaria leaf spot on soybean in relation to weather parameters in different dates of sowing under epiphytotic conditions during *Kharif*, 2019.

S. No	Standard week	Meteorological weeks	Temperature (°C)		Relative humidity (%)		Rainfall (mm)	AUDPC*			
			Max	Min	Max	Min		Inoculated on			
								8th July	15th July	22th July	29th July
1	28	9–15 July 2019	31.8	24.0	71.6	57.8	0.0	0.00	0.00	0.00	0.00
2	29	16–22 July 2019	33.6	23.7	76.9	55.0	34.6	32.94	0.00	0.00	0.00
3	30	23–29 July 2019	31.5	24.1	81.0	67.6	12.6	73.33	30.66	0.00	0.00
4	31	30-5Aug. 2019	30.6	22.9	88.4	78.6	15.0	91.74	66.64	26.39	0.00
5	32	6–12 Aug. 2019	28.7	21.9	87.1	78.9	182.2	107.00	79.73	59.64	28.53
6	33	13–19 Aug. 2019	27.6	21.6	90.3	80.0	153.0	119.77	94.40	73.15	63.53
7	34	20–26 Aug. 2019	31.7	21.3	82.9	63.7	69.6	135.80	109.45	86.10	75.25
8	35	27–2 Sept. 2019	30.0	21.7	94.3	78.4	141.0	166.60	123.24	101.50	86.45
9	36	3–9 Sept. 2019	29.3	22.8	92.7	84.4	85.5	227.96	136.19	116.73	97.34
10	37	10–16 Sept. 2019	30.6	22.4	87.9	77.3	36.1	269.54	152.08	129.68	108.89
11	38	17–23 Sept. 2019	31.8	21.0	87.7	62.4	42.2	292.15	181.37	151.06	130.41
12	39	24–30 Sept. 2019	29.4	21.8	92.4	73.3	14.2	320.04	214.87	181.20	161.49
13	40	1–7 Oct. 2019	30.2	18.6	89.1	68.0	66.4	334.50	267.37	211.51	188.62
14	41	8–14 Oct. 2019	31.5	16.3	85.6	62.1	18.4	349.72	315.49	239.82	210.25
15	42	15–21 Oct. 2019	32.0	16.1	80.1	46.3	0.0	370.27	333.45	275.45	234.89
16	43	22–28 Oct. 2019	29.1	13.2	80.6	38.1	0.0	394.42	354.06	312.03	259.18

*Mean of three replications, Inoculation started 21 days after sowing of each date. *Observation started 7th days after inoculation and at weekly intervals, 1st sown-18th June, 2nd–25th June, 3rd–02th July, 4th–09th July 2018.

**Table 7:** Effect of sowing dates on grain yield of soybean (cv. RKS-24) during *Kharif* 2018 and 2019.

Date of sowing	Seed yield (kg ha^−1^) 2018	Seed yield (kg ha^−1^) 2019	Pooled
18th June	1461.11	1421.29	1441.20
25th June	1510.18	1485.18	1487.68
02th July	1581.48	1664.81	1623.14
09th July	1715.74	1683.33	1699.53
CD at 5%	175.86	201.28	119.00
CV%	5.62	6.45	6.04

*Mean of three replications.

## Discussion

**Figure 3:**
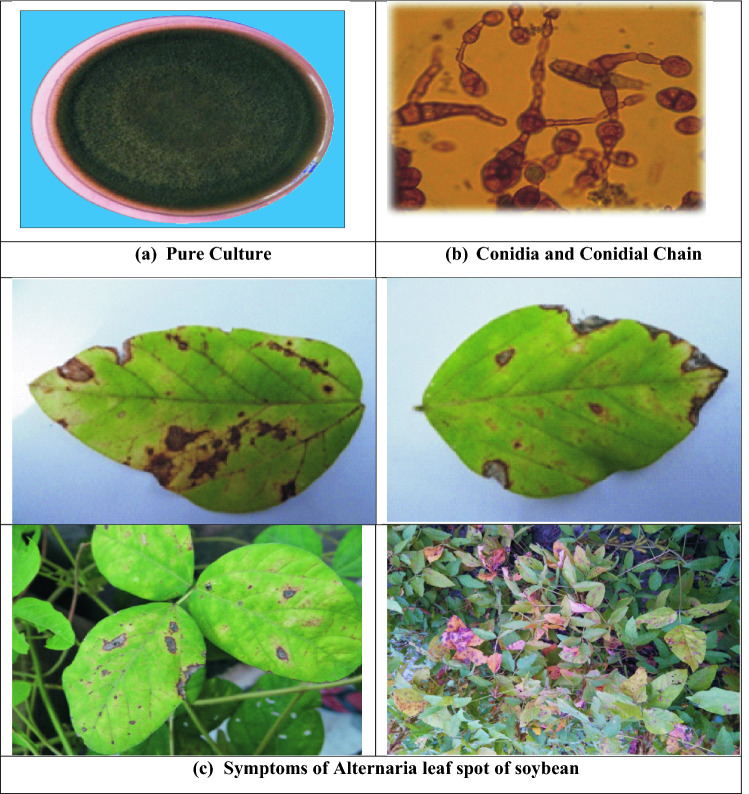
Characteristics and symptoms of *A. alternata.*

**Figure 4:**
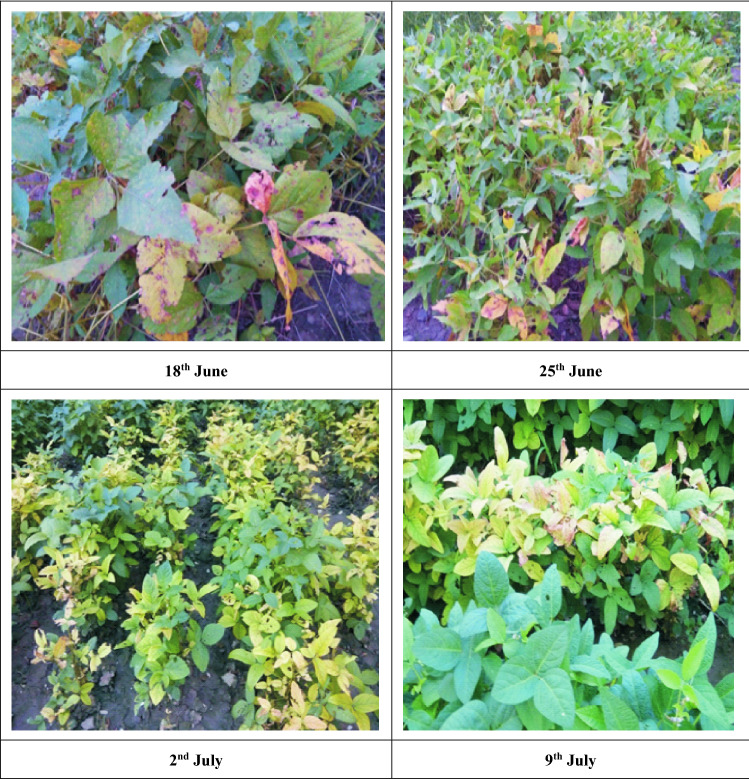
Effect of different date of sowing on development of Alternaria leaf spot soybean in field conditions.

Among foliar diseases, Alternaria leaf spot is common in soybean growing countries and was first reported from Antalya Province of Turkey^14^. Disease prevalent in moist warm humid areas and the severity of the disease depends on environmental conditions and susceptibility of common cultivars^15^. Infected leaves develop brown necrotic spots with concentric rings that appear on foliage. These spots coalesce and form large necrotic areas in advanced stages. Infected leaves eventually dry out and drop prematurely and yellow halo was recorded around the spots on leaves^15^ (Fig. 3c), cause significant damage up to 57%^16^. Before conducting field experiments the infected sample of soybean collected for isolation of pathogen after purification and identified as *A. alternata* for further studies (Fig. 3a,b). The disease development under field conditions was influenced by various environmental factors, host cultivar and availability of inoculums load. Weather factor and staggered sowing dates played an important role in the severity of Alternaria leaf spot of soybean, which governed the variability in onset of the disease and epidemics development. In present study, per cent disease severity periodically increased with the time in all dates of sowing. The progression of disease was slow during the period between 16th July and 2nd September among all dates of sowing. In this period, the minimum and maximum temperatures were 22.0–32.2 °C and 21.3–33.6 °C accompanied with relative humidity 74.1–83.7% and 55.0–94.3% with 47.07 and 76.0 mm average rainfall. Following this, the disease severity increased gradually. There was sharp increase in disease severity observed during 24th September–28th October in all dates of sowing during both the years. During this period, the minimum and maximum the temperature were 18.1–34.4 °C and 13.2–32.0 °C accompanied with minimum and maximum relative humidity 23.0—80.4% and 38.1–92.4% with 5.16 and 19.8 mm average rainfall during both the years, respectively (Figs. 1 and 2). The weather conditions during this period might have created the congenial conditions for severe infection of the pathogen. In previous study, the maximum temperature of 27–28 °C minimum temperature of 14–15 °C and average relative humidity more than 65% was found favourable for Alternaria blight development of Indian mustard^17^. Multiple regression equation and coefficient of determination value indicate that the temperature and relative humidity reflected during this period maximum temperature range was 14.0–38.0 °C and minimum temperature 6.0–21.0 °C while maximum relative humidity 54–93% and minimum relative humidity 20–68% contribute 84–100% in early blight development in tomato (Fig. 4)^18^.

In present study, the disease severity was positively correlated with maximum temperature, while it is negatively correlated with minimum temperature, minimum and maximum relative humidity and rainfall for all dates of sowing of *Kharif,* 2018 are presented in Table 1. The disease severity was negatively correlated with minimum temperature for all dates of sowing. But disease severity was negatively correlated with maximum, minimum relative humidity and rainfall in crop sown as 2nd July and 9th July, 2019 (Table 4). The disease severity of Alternaria blight of Indian mustard was positively correlated with maximum and minimum temperature, morning and evening average vapour pressure, wind speed, sunshine hours and evaporation, while it was negatively correlated with morning and evening relative humidity^13^.

In present study, the maximum AUDPC was recorded as 389.45 and 394.42 in 18th June sown crop. However, minimum AUDPC of 266.18 and 259.18 was recorded on 9th July sown crop (Tables 3 and 6). It has previously been proposed that Alternaria blight AUDPC values were low during 22nd July–3rd September when temperature ranges in 22.8–29.1 °C and relative humidity 76.1–89.3%. The AUDPC values were moderate during 10th September–30th September 2014. While increase in temperature (20.0–33.9 °C and 18.4–31.4 °C) during 1st October to 6th November 2014, the disease progression was much faster and high AUDPC values were obtained. Alternaria blight severity increased with increasing in temperature (19.8–32.5 °C) & Sunshine hours, decreasing in rainfall & RH during 17th Sep.–6th Nov. period, the disease progression was much faster and AUDPC values of 206.1–378.3 were observed in cluster bean^19^. Results of present study, the maximum seed yield were recorded in 9th July sown crop and minimum seed yield was recorded in 18th June sown crop during both years (Table 7). In previous study, the minimum interaction effect between date of sowing and maximum seed yield (1886.18 kg/ha^−1^) obtained in early sown crop and minimum 1685.59 kg/ha^−1^ recorded in late sown crops. Highest disease intensity of Alternaria blight in linseed was recorded when crop was sown on 10th October and it gradually declined on later dates sown crop. Highest seed yield with less disease intensity was obtained when crop was sown on 30th October followed by 20th October^20^.

Moisture and temperature can not be considered separately. Decreased levels of rainfall may lead to decreased disease incidence. However, in a warming scenario, the increase in temperature more than compensates for the reduction in duration of leaf wetness, in part because infections that start earlier in the growing season allow more time for epidemics to develop^21^. Although increased temperatures are generally associated with an increased risk of disease development for most pathosystems, in some cases decreased precipitation results in a decreased risk of disease^22^. Environmental changes can also indirectly influence the biology of a pathogen by changing the plant architecture, thereby altering the microenvironment. The canopy density and structure can affect the temperature and moisture at an infection site. Increased plant and leaf densities tend to increase leaf wetness, thus promoting the development of pathogens that prefer humid conditions^23^. Temperature and moisture govern the rate of reproduction of many pathogens^24^. The longer growing seasons that will result from global warming will extend the amount of time available for pathogen reproduction and dissemination. Climate change may also influence the sexual reproduction of pathogens^25^.

## Conclusion

Weather conditions play a predominant role in determining the course and severity of epidemics. Hence, to study the role of different weather parameters viz*.,* temperature, relative humidity and rainfall are most important factors for infection and progression of Alternaria leaf spot of soybean. It severity increased with increase in temperature (15.1–34.4 and 13.2–32.0 °C) and decrease in rainfall & RH during 24th September to 28th October period, the disease progression was much faster with disease severity (57.82% and 58.22%) and AUDPC (389.45 and 394.42) recorded in crop sown on 18^th^ June during both of the year.

## Materials and methods

### Sample collection

The diseased sample was collected from major soybean growing areas of Rajasthan province of India during *Kharif* season (July–September) of 2018. The infected plant parts (leaves) of the diseased samples were carefully placed in polythene bags (20 μm), tagged and brought in the Department of Plant Pathology (12 h.) at Rajasthan College of Agriculture, Udaipur. Fungal infected leaves were examined and used for the pathogen isolation. The plant material used in the study is widely cultivated variety at farmer’s field and collection of sample undertaken the guideline of Government of India, and there is no ethical issue.

### Preparation of media, isolation and purification of the pathogen

Potato dextrose agar (PDA) plate method was used for fungal isolation. The medium was prepared by adding peeled potato (200 g), dextrose (20 g) and agar (15 g) per litre. The pH of medium was adjusted (6.5) using citrate phosphate buffer solution and pH was corrected using digital pH meter (ELICO LI 120). After that prepared media were placed in flask (1000 ml) and transferred to for sterilization. The sterilized media (15–20 ml) were transferred in each Petri plates in the laminar air flow (MICRO-FLIT (India), and kept for 15–20 min to condense media for further use in isolation. Diseased sample cut into small pieces (2–5 mm) with sterilized scalpel, washed with ultra-purified water (0 TDS), and dip in 0.1% mercuric chloride (HgCl_2_) solution for 2 min. The pieces were again washed with ultra-purified water (0 TDS) in triplicates. The diseased sample were transferred into already prepared condense media (PDA) in Petri plates under aseptic conditions. Petri plates were transferred to BOD (PSI-4) and incubated at 26 ± 1 °C for pathogen mycelial growth. After 3–4 days, peripheral growth of mycelium was appeared and picked up mycelial growth with sterilized inoculation needle for sub culturing of pathogen in similar condition. The culture was purified by single spore techniques described by Sofi et al*.*^26^.

### Identification of the pathogen

The pathogen was identified as *A. alternata* on the basis of cultural and morphological characteristics seen in isolated pathogen. The identified pathogen was subculture in a PDA slant and sent to Indian Type Culture Collection (ITCC), Division of Plant Pathology, Indian Agriculture Research Institute (IARI), New Delhi, for confirmation. Dr. T. Pramila Devi, Principal Scientist confirmed the pathogen as *A. alternata* and was deposited with ITCC ID No. 10.810.18, 2018.

### Inoculum preparation

The conidial suspension was prepared using standard method previously described by Boedo et al*.*^27^. Fungal cultures were flooded with sterile water and conidia dislodged gently with glass plate, filtered (mycelial and conidial suspension) with two layers cheesecloth and counted spore density using haemocytometer and adjusted to 1 × 10^3^ conidia ml^−1^.

### Field experiments for disease assessment

The field experiments were conducted in the *Kharif* season of 2018 and 2019 at Experimental field, Rajasthan College of Agriculture, MPUAT, Udaipur (longitude- 73° 42′ 54.96″ E and latitude- 24° 33′ 24.58″ N) in sandy-clay loam soil (sand 39.49, silt 26.94 and clay 33.57%). The available NPK of soil was, 301 kg, 18.5 kg, 580 kg per ha., respectively. The pH and electrical conductivity of soil was 7.5 and 0.98 dSm^−1^, respectively, with 0.58% soil organic carbon under irrigated condition. The experiment was laid out under randomized block design (RBD) with three replications. A susceptible cultivar RKS-24 sown in staggered sowing dates from 18 June to 9th July in 7 d intervals in triplicates. The planting was done in plot size of 3.0 × 3.6 m^2^ and R × P (30 × 10 cm) with ten rows per plots per replications. Plants were inoculated (as of 21 days) using battery operated knapsack sprayer with a spore suspension of *A. alternata* 1 × 10^3^ conidia ml^−1^ on 8th July, 15th July, 22th July and 29th July of both the year for disease development in soybean plants.

### Observation recorded

Per cent disease index (PDI) from initiation of disease and at 7 d interval was calculated up to maturity, using 0–5 standard disease rating scale proposed by Sangeetha and Siddaramaiah (2007)^17^. Results were calculated using formula suggested by Chester^28^ and Wheeler^29^ described below.

$$PDI = \frac{n \times 1 + n \times 2 + n \times 3 + n \times 4 + n \times 5}{N} \times \frac{100}{{Maximum\, disease\, score (5)}}$$

where n = Number of plants in each score, 1–5 = disease score, N = Total number of plants under observation.

According to observed PDI, area under disease progress curve (AUDPC) was calculated. Weather parameters such as temperature, relative humidity and rainfall were recorded during entire crop period and correlation was worked out. To know the relationship between the dependent (PDI) and independent variables (max. temp., min. temp., max. RH, min. RH and rainfall), the analysis of multiple regression was done from 18 June to 9th July of 2018 and 2019 on weekly basis.

Weekly meteorological data used in experiment were obtained from Agro-met observatory, RCA, Udaipur to establish correlation with disease development. Multiple regression, correlation coefficient and coefficient of multiple determination (R2) were calculated as per the standard statistical formula Y = a + b1X1 + b2X2 + b3X3 + b4X4 + b5X5. Where, Y = PDI, a is intercept/ constant value, b1… b5 are regression coefficient of corresponding independent weather variables, X1…X5 are independent weathers variables^30^. AUDPC was calculated as formula suggested by Campbell and Madden^12^ as follow:

$$AUDPC\, = \,\left[ {\left( {\frac{{X_{i + 1} \, + \,X_{i} }}{2}} \right)\,\, \times \,\,(t_{i + 1\,} - \,\,t_{i} )} \right]$$

where Xi = The cumulative disease index expressed as proportion at the *i*th observation, ti = Time (days after planting) at the *i*th observations. n = Total number of observations.

### Statistical analysis

Study was performed in randomized block design (RBD) with three replications. The ANNOVA for all the weather parameters and disease severity were statistically performed with software package of SPSS 18.0 for window (SPSS inc, Chicago, IL, USA was used. Multiple regression and correlation was performed at 0.05 and 0.01 level of significance.
